# 2559. Antibiotic-induced Bartter-like syndrome - A Systematic Review

**DOI:** 10.1093/ofid/ofad500.2176

**Published:** 2023-11-27

**Authors:** Megha Priyadarshi, Saurav Sekhar Paul, Manish Soneja

**Affiliations:** All india institute of medical sciences, Delhi, Delhi, India; All india Institute of Medical Sciences, New Delhi, delhi, Delhi, India; All India Institute Of Medical Sciences, Delhi, Delhi, India

## Abstract

**Background:**

Bartter syndrome encompasses salt-losing tubulopathies characterized by hypokalemia, hypochloremic metabolic alkalosis, and hyperreninemic hyperaldosteronism with normal blood pressure. Acquired Bartter or Bartter-like syndrome (BLS) is commonly associated with the use of diuretics and antibiotics such as capreomycin, netilmicin, colistin, and amphotericin B. This systematic review was done to understand the time of occurrence of Bartter syndrome post-inciting drug and gain knowledge regarding its progression and management.

**Methods:**

We searched PubMed, Google Scholar, Embase, medRxiv, and bioRxiv databases for articles published from 1986 to March 2022 using the keywords "Antibiotic," "Bartter Syndrome," "Pseudobartter Syndrome," and "Bartter-like." Studies that reported cases of BLS in adults or children with confirmation of the inciting drug were included. We excluded studies that did not provide sufficient details of the clinical presentation and outcome.

**Figure:**
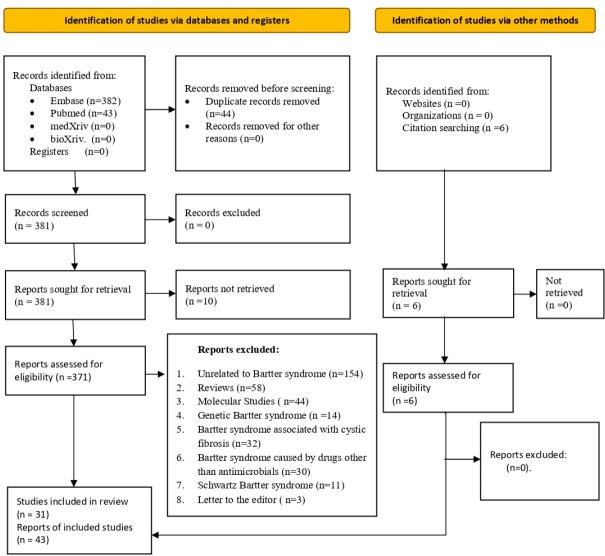

**Results:**

We identified 31 studies, comprising 43 cases of BLS. The mean age of the patients was 41.1 years (range: 25–57 years), and 65.1% were female. The most common antimicrobial class associated with BLS was aminoglycosides (65.1%), followed by the polymyxin group. The most common antimicrobial was Gentamicin (41.8%), followed by Colistin (32.5%), Streptomycin, and Capreomycin (6.9% each). Hypokalemia(100%), metabolic alkalosis(97.2%), hypocalcemia(92.5%), and hypomagnesemia(100%) were reported in almost all patients. The median time of onset after exposure to the inciting agent was 10 days (range 7–18 days), and the median time for resolution after stopping the drug was 14 days (range 7–31 days). There was a complete resolution of the symptoms following discontinuation in all cases.

**Figure:**
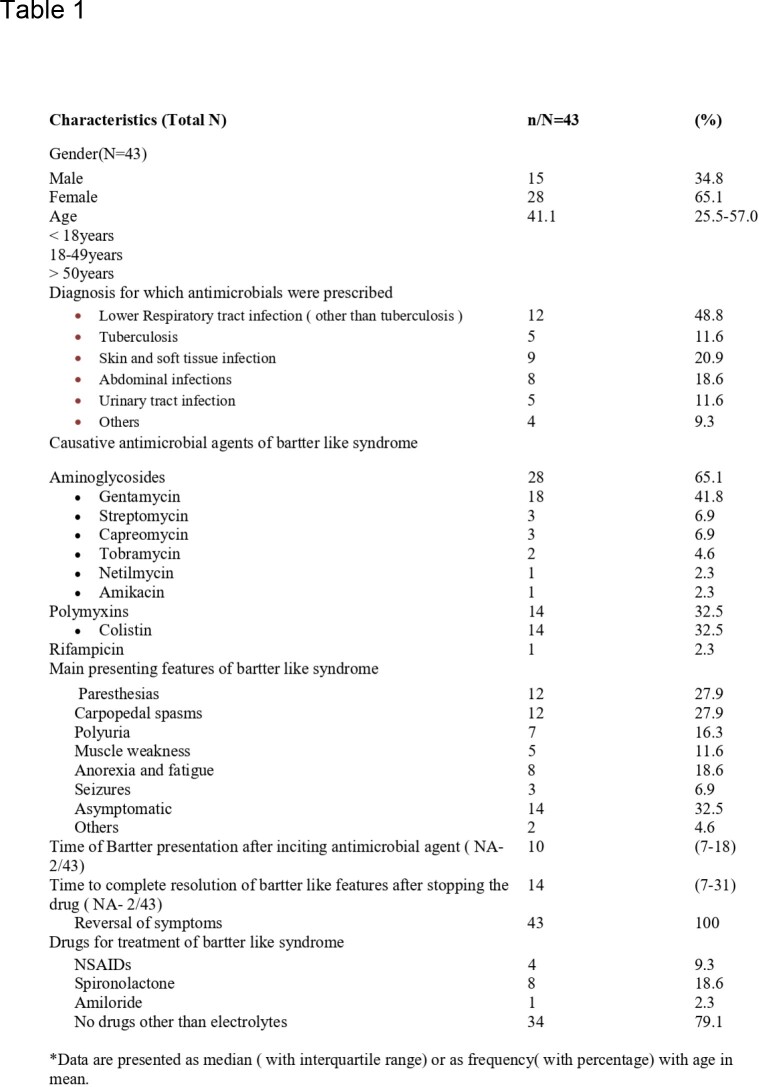

Demographic and baseline characteristics of the patients

**Figure:**
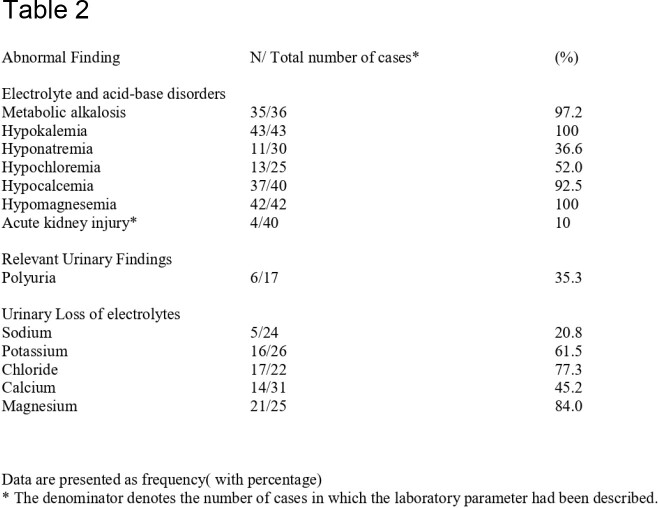

Prevalence of electrolyte, acid-base disorder, and urinary electrolyte abnormalities

**Figure:**
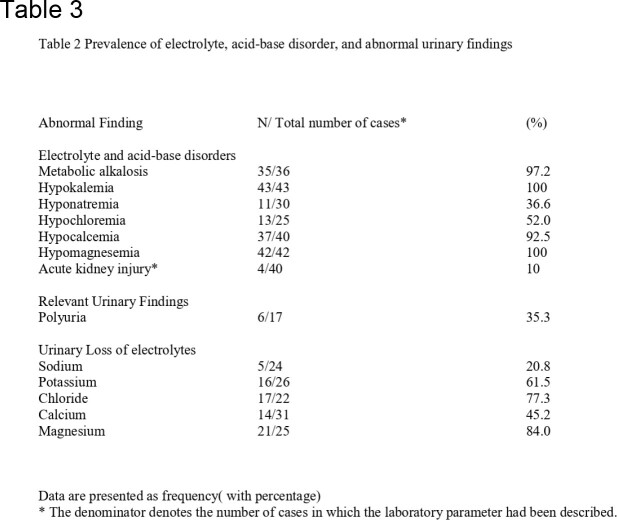

Laboratory parameters among the reported cases

**Conclusion:**

BLS is a rare but potentially reversible complication of various antimicrobials, most commonly aminoglycosides. The diagnosis requires a high index of suspicion and a careful review of the drug history. The prognosis is favorable, with a complete reversal of the state after drug discontinuation.

**Disclosures:**

**All Authors**: No reported disclosures

